# Multi-parametric effect in predicting tumor histological grade by using susceptibility weighted magnetic resonance imaging in tongue squamous cell carcinoma

**DOI:** 10.1186/s12880-019-0322-8

**Published:** 2019-03-12

**Authors:** Xing Yang, Jinyu Zhu, Yongming Dai, Zhen Tian, Gongxin Yang, Huimin Shi, Yingwei Wu, Xiaofeng Tao

**Affiliations:** 1 Department of Radiology, Shanghai Ninth People’s Hospital, Shanghai Jiaotong University, School of Medicine, Shanghai, 200011 China; 2 United Imaging Healthcare, Shanghai, 201807 China; 3 Department of Pathology, Shanghai Ninth People’s Hospital, Shanghai Jiaotong University, School of Medicine, Shanghai, 200011 China

**Keywords:** MRI, Susceptibility-weighted imaging (SWI), Oral tongue squamous cell carcinoma (OTSCC), Intratumoral susceptibility signal intensities (ITSSs)

## Abstract

**Background:**

Susceptibility weighted imaging (SWI) is helpful for depicting hemorrhage, calcification, and increased vascularity in some neoplasms, which may reflect tumor grade. In this study, we aimed to apply SWI in patients with oral tongue squamous cell carcinomas (OTSCCs) and relate multi-parametric effect to tumor histological grade prediction.

**Methods:**

Preoperative MR examinations were performed on a 1 .5T MRI scanner with T1-, T2- and contrast-enhanced (CE) T1-weighted imaging. In addition to routine head and neck MRI sequences, SWI was performed. Tumor thickness and volume were measured. Intratumoral susceptibility signal intensities (ITSSs), ITSS score and ITSS ratio on SWI were evaluated and recorded. Subjects were sub-grouped into low- and high-grade according to the histological findings post operation. Parameters such as tumor thickness, tumor volume and three ITSS related parameters were compared between low- and high-grade groups. ROC analysis was performed on above parameters to access the capability in predicting tumor histological grade. Different multi-parametric models were run to access multi-parametric combination effect.

**Results:**

Thirty patients with OTSCC were finally included in the study. Twenty of them were categorized as low-grade SCC and the other ten subjects were high-grade SCC according to the pathologic findings. No significant difference was seen for tumor thickness or tumor volume between two sub-groups. ITSSs were seen in 23/30 patients. Significant difference of ITSS scores between low- and high-grade OTSCCs was observed, with mean value of 0.95 ± 0.83 and 1.70 ± 0.95, respectively. Univariate ROC analysis demonstrated ITSSs, ITSS score and ITSS ratio were valuable parameters for predicting tumor histological grade and ITSSs was superior to the other two parameters, with an area under ROC curve of 0.790. Multi-parametric model using combination of ITSSs and tumor thickness would greatly improve the predictive capability in comparison with a univariate approach, yielding the area under ROC curve of 0.84(0.69,0.99). On contrast-enhanced SWI (CE-SWI), ITSSs were shown more clearly delineated in comparison with non-contrast enhanced SWI.

**Conclusions:**

In conclusion, SWI was superior in depiction of internal characteristics of OTSCCs, which would potentially provide more diagnostic information. Multi-parametric model using combination of ITSSs and tumor thickness would be valuable in predicting tumor histological grade.

## Background

Oral tongue cancer is the most common malignancy of oral cavity, and accounted for approximately 30–40% of oral cavity cancer (OCC) [1–3]. OTSCC still has an unfavorable prognosis with a 5-year survival rate of around 50% [4–6]. Typical anatomical characteristics of the oral tongue result in no barrier existing for preventing tumor propagation. Furthermore, abundant lymphovascular system around the oral tongue makes cervical metastasis more prone to occur. Magnetic resonance imaging (MRI) has been considered the most valuable imaging modalities in tumor detection and tumor grade evaluation as well [7–9]. With the measurement of primary tumor dimensions, the assessment of tumor prognostic factors and the observation of cervical metastasis, MRI could be helpful in tumor TNM (Tumor Node Metastasis) stage determination. Many studies reported that conventional TNM staging system had some limitations in prediction of outcomes of OTSCC whereas some clinicopathological parameters such as tumor thickness (TT) and depth of invasion (DOI) might be better predictors for patient prognosis [10–12]. Therefore, it is desired to figure out more parameters or characteristics of OTSCC on preoperative radiographs for further appropriate treatment [13, 14].

MRI is regarded as the preferred imaging modality in diagnosis of oral area diseases [15, 16]. However, there still exist difficulties in distinguishing the malignancy grade or tumor stage of oral tongue cancer with routine head and neck MRI sequences. SWI is an imaging technique using the intrinsic nature of local magnetic fields to enhance image contrast to improve the visibility of various susceptibility sources and to facilitate diagnostic interpretation [17]. SWI provides complementary information on venous vasculature, hemorrhage, calcification or iron deposition since they have both magnitude and phase shift to enhance susceptibility changes [17, 18]. Therefore, SWI is helpful in neuroimaging and cancer imaging to detect more internal characteristics. Nowadays, SWI has been widely used in differential diagnosis of central system tumors and assessing WHO grade for brain astrocytomas [19–24]. Intratumoral various characteristics such as vasculature and microhemorrhage may reflect tumor heterogeneity and be related to tumor histological grade. Numerous studies emphasized capability of SWI in detection of vasculature and microhemorrhage in brain tumors such as gliomas [20, 21]. Semi-quantitative measurement of ITSSs on SWI has been reported as a promising parameter for both tumor grade evaluation and central nervous system lymphoma and atypical glioblastoma differentiation [22–25]. Besides application in central nervous system, SWI has shown the ability to improve siderotic nodules detection in cirrhotic liver [26]. Moreover, SWI application in patients with renal cell carcinoma proved to be helpful in identifying hemorrhage and predicting tumor nuclear grade [27, 28].

To date, however, SWI has not yet been applied in OTSCC. Therefore, the aim of this explorative study is to apply SWI in patients with OTSCC and relate multi-parametric combination to tumor histological grade prediction.

## Methods

### Subjects

The prospective study was approved by institutional review board. Total 37 patients were consecutively recruited in the study from August 2016 to March 2017. Eligible subjects were enrolled with the following criteria: a) Patients with suspected oral tongue cancer (both clinical and biopsy suspected) planned to have surgical treatment; b) None biopsies, radiotherapy, chemotherapy or surgical treatment was performed prior to MR examination; c) Oral cavity cleaning was required before MR examination to avoid the effects caused by food residues; d) No metal denture or swallowing artifacts during the examination.

### MR imaging protocol

All subjects were scanned on a 1 .5T MRI system (United Imaging Healthcare, Shanghai, China) with a commercial twelve-channel head-neck joint coil. SWI was requested in addition to conventional head and neck MR imaging. The protocol of MRI for all the patients included: (a) transverse fat-suppressed fast-spin-echo(FSE)T2-weighted imaging (T2WI) (TR/TE, 4000/72.1 ms; matrix size, 304 × 304; slice thickness, 4 mm; flip angle, 150°); (b) transversal T1-weighted imaging (T1WI) FSE (TR/TE, 500/11.84 ms; matrix size, 304 × 304; slice thickness, 4 mm; flip angle, 150°); (c) coronal T2WI FSE (TR/TE, 3800/96.66ms; matrix size, 320 × 320; slice thickness, 3 mm; flip angle, 150°); (d) FSE Contrast-enhanced-T1WI (TR/TE, 450/9.96 ms; matrix size, 304 × 304; slice thickness, 4 mm; flip angle, 150°). (e) 3D-GRE SWI (TR = 25 ms; TE = 18 ms; field of view, 220 mm × 220 mm; matrix size, 320 × 320; slice thickness, 2 mm; flip angle, 18°; acquisition time 3:40 min). Gadopentetate dimeglumine (Magnevist, Schering, Berlin, Germany) was injected via a power injector with a flow rate of 2.0 ml/s at the dose of 0.1 mmol/kg of body weight after coronal T2WI sequence.

Axial scan sequences order was T2WI, T1WI and then CE-T1WI. SWI was performed before contrast-enhanced scan, five subjects were performed with SWI both pre- and post-contrast-enhanced scan, and the ‘locate the center slice’ mode on the operation workstation was chosen in order to match the slices.

### Image processing and analysis

All image assessments were performed on uMR560 workstation of United Imaging independently by two radiologists with over 5 years’ experience. They were blinded to either patient medical history or pathological results. Lesion size, location, margin, signal intensity and tumor thickness were observed and recorded on routine head and neck MRI images. Tumor thickness was measured by counting number of axial T2WI slices where the lesion was visible and multiplying the number of axial T2WI slices by slice thickness. After the lesion involving area in each slice was delineated with an operator-defined ROI, tumor volume was obtained by the sum of lesion area (in each slice) × slice thickness by Image J software (NIH, Bethesda, USA). All quantitative measurement was performed three times and the mean value was recorded for further analysis. Qualitative measurement was recorded independently by above two experienced radiologists.

SWI images of all 30 patients were acquired and evaluated by two experienced radiologists. First of all, we selected an axial section visually showed the maximum frequency of the ITSS within a tumor. ITSSs were counted on this axial section and then ITSS score was evaluated according to previous studies [29]. Intratumoral calcifications, tumor necrosis tumor or macrohemorrhages have been excluded on routine MRI sequences and diffusion-weighted images. Tumor necrosis was differentiated as areas with hyper-intense signal on T2-weighted images and the interior non-enhancing part of an enhancing lesion on contrast-enhanced axial images. ITSSs were defined as low signal intensity and a fine linear or dot-like structure seen within the tumor on SWI, with or without conglomeration [29]. ITSS score was divided into four grades: Score 0 indicated absence of ITSS; Score 1 indicated less than 5 ITSSs; Score 2 indicated 6–10 ITSSs; and Score 3 indicated more than 10 ITSSs [29]. ITSSs and ITSS score were accessed and compared between high- and low-grade groups. Pathologic reports were made in accordance with WHO (2005) criteria (grade I-III). For statistic purpose, OTSCCs were divided into low-grade (WHO grade I and I-II), and high-grade (WHO grade II and II-III). Regarding the effect of tumor size on ITSS score, ITSS ratio was established in this study. ITSS ratio was defined as the ratio of ITSSs to the lesion involving area in tumor maximal axial section.

Contrast-to-noise ratio (CNR) was measured and compared on pre- and post-contrast SWI images. The CNR was calculated using the following formula.

$$ \left({\mathrm{SI}}_{\mathrm{tumor}}-{\mathrm{SI}}_{\mathrm{normal}\ \mathrm{tissue}}\right)/{\mathrm{SD}}_{\mathrm{background}} $$

SI _tumor_ is the mean signal intensity in the region of interest (ROI) in the area of the tumors; SI _normal tissue_ is the normal-appearing tissue surrounding the tumor; and SD _background_ is the standard deviation of the background noise in the frequency-encoding direction.

### Statistical analysis

All the statistics were analyzed by SPSS 20.0 software (IBM, Armonk, New York, USA). Tumor volume or tumor thickness comparison between low- and high-grade of squamous cell carcinomas (SCCs) was performed by non-parametric Mann-Whitney test, and it was also used to compare the differences of ITSS score on SWI between low- and high-grade tumors. For single parameter, the capability in predicting tumor histological grade was quantified by the area under the curve of the receiver operating characteristic (ROC) analysis. Then multi-parametric models including two parameters were generated. ROC analysis was performed and ROC curve was drawn by Medcalc 12.7.0 (MedCalc Software, Ostend, Belgium). *P* < 0.05 was considered statistically significant. Inter-rater reliability for dichotomous and classified variables (ITSS score) was determined by using the Cohen k coefficient. Intra-class correlation coefficient was calculated for continuous variables (tumor thickness, tumor volume, ITSSs). Interpretation for Cohen k coefficient and intra-class correlation coefficient was according to methods described by Landis and Koch as following [30]. Less than 0, no reproducibility; 0.0–0.20, slight reproducibility; 0.21–0.40, fair reproducibility; 0.41–0.60, moderate reproducibility; 0.61–0.80, substantial reproducibility; and 0.81–1.00, almost perfect reproducibility.

## Results

### Cohort characteristics

Total 30 patients (8 female, 22 men, mean age 56.2 ± 11.4 yrs) were prospectively recruited in the study (7 out of 37 cases were excluded due to the serious artifacts). All subjects were histologically diagnosed with SCCs post operation. Twenty of them were categorized as low-grade SCCs and the other ten subjects were high-grade SCCs according to the pathologic findings (Table 1). Most of cases (22/30, 73.3%) manifested as single marginal lesion at the edge of the tongue. Routine head and neck MRI showed T1WI hypointense and T2WI hyperintense lesion with obvious enhancement post contrast administration.

**Table 1:** Cohort characteristics

Patient	Gender	Age	WHO Grading	Pathological Grading	Tumor Volume (cm^3^)	Tumor Thickness (mm)	ITSSs (n)	ITSS Score
1	F	66	I	low	18.7	35	12	3
2	F	66	I	low	1.3	12	0	0
3	M	70	I	low	50.1	55	4	1
4	M	55	I	low	17.8	32	3	1
5	M	52	I	low	27.4	36	7	2
6	M	20	I	low	3.9	12	8	2
7	M	50	I	low	35.2	34	3	1
8	M	62	I-II	low	1.8	24	2	1
9	M	66	I-II	low	8.6	24	2	1
10	M	57	I-II	low	41.4	44	8	2
11	M	49	I-II	low	1.6	18	0	0
12	F	53	I-II	low	4.7	18	3	1
13	M	51	I-II	low	1.6	12	2	1
14	M	40	I-II	low	1.9	12	2	1
15	F	80	I-II	low	0.5	4	0	0
16	M	59	I-II	low	4.8	20	5	1
17	M	61	I-II	low	2.5	12	0	0
18	F	69	I-II	low	0.4	6	0	0
19	M	59	I-II	low	2.2	12	0	0
20	M	47	I-II	low	4.4	22	2	1
21	M	53	II	high	23.3	38	9	2
22	M	51	II	high	5.6	16	5	1
23	M	56	II	high	4.5	18	3	1
24	F	76	II	high	16.1	36	12	3
25	M	61	II	high	3.1	18	8	2
26	M	56	II	high	3.7	18	9	2
27	M	49	II	high	4.8	20	13	3
28	F	54	II	high	4.9	18	6	2
29	F	55	II-III	high	11.1	12	0	0
30	M	42	II-III	high	2.8	20	5	1

Note. ITSS score was divided into 0–3: Score 0 indicated absence of ITSS; Score 1 indicated less than 5 ITSSs; Score 2 indicated 6–10 ITSSs; and Score 3 indicated more than 10 ITSSs

### SWI results

**Fig. 1:**
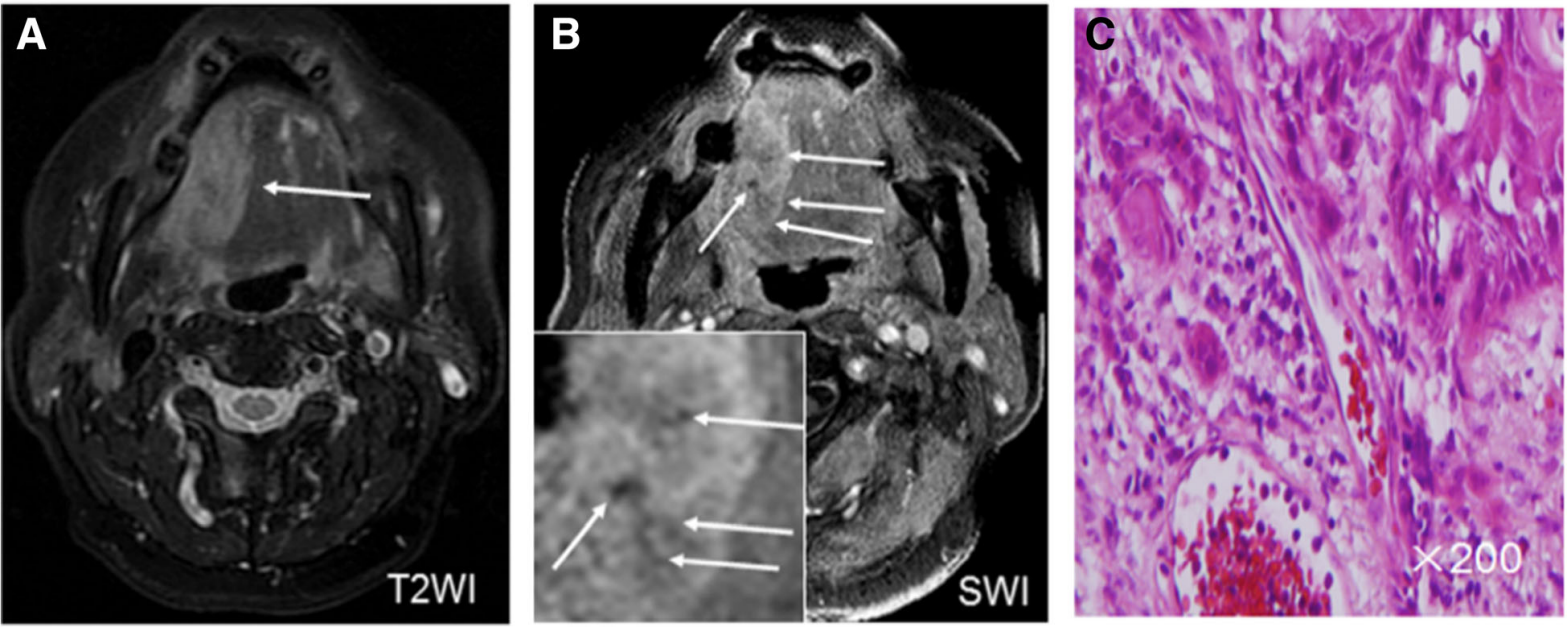
A histological SCC on right oral tongue (**a**) Hyperintense signal of the tumor lesion was observed on T2WI at the edge of right oral tongue (arrowhead). **b** SWI showed multiple linear-shaped or dot-like ITSSs (arrowhead) within the lesion. **c** Histological findings showed multiple vessels in the corresponding site of the tumor

**Fig. 2:**
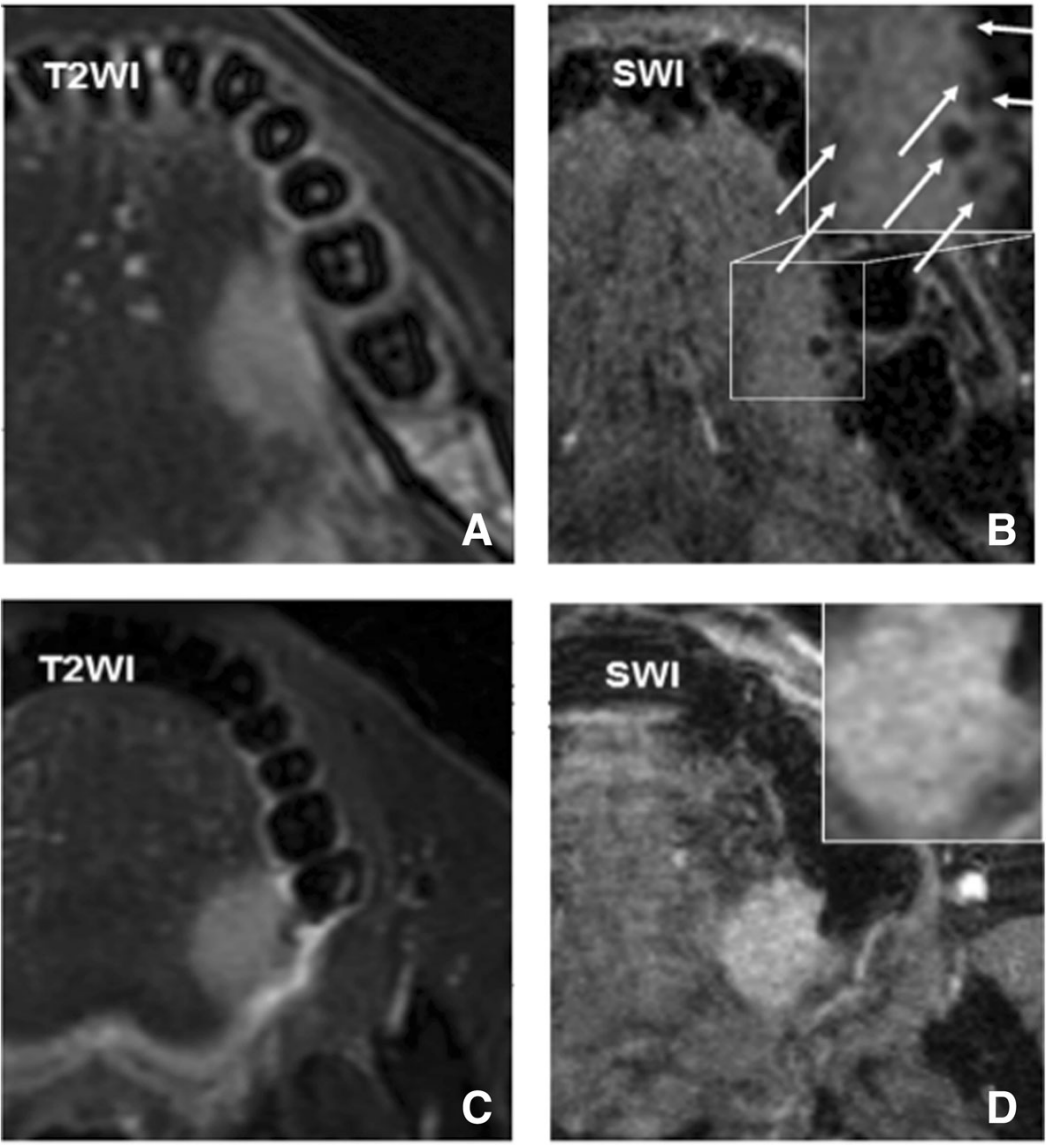
**a**-**b** A histological high-grade SCC on left oral tongue (**a**) Hyperintense lesion was shown on T2WI (**b**) At least 5 ITSSs was seen within the tumor (arrowhead) and its ITSS score was defined as 2. **c**-**d** A histological low-grade SCC on left oral tongue (**c**) Hyperintense lesion was shown on T2WI. **d** No apparent ITSS was seen within the tumor and therefore its ITSS score was defined as 0

Of the 30 tumors, ITSSs were seen in 23(76.7%) cases (Fig. 1). The presence of ITSSs in low-grade group and high-grade group was 14(70%) and 9(90%), respectively. ITSS scores distribution in high-grade group were 0 points (10%), 1 points (30%), 2 points (40%) and 3 points (20%) ITSS score for low-grade group were 0 points (30%), 1 points (50%) 2 points (15%), and 3 points (5%)(Table 1). Mean ITSS scores for low- and high-grade SCCs were 0.95 ± 0.83 and 1.70 ± 0.95, respectively (Table 2 and Fig. 2). ITSS scores were significantly lower in low-grade than in high-grade tumors (*P* < 0.05).

**Table 2:** Comparison of single variables between low- and high-grade tumors

Variable	Low-grade	High-grade	*P* value	*Z* value
Mean ITSS score	0.95 ± 0.83	1.70 ± 0.95	0.036	−2.095
ITSS scores, n(%)	20(100%)	10(100%)		
0	6(30%)	1(10%)		
1	10(50%)	3(30%)		
2	3(15%)	4(40%)		
3	1(5%)	2(20%)		
Tumor thickness(mm)	22.2 ± 13.4	21.4 ± 8.5	0.812	
Ln [Tumor volume (cm^3^)]	1.5 ± 1.4	1.8 ± 0.7	0.5

### Comparison of tumor thickness and tumor volume between low- and high-grade SCCs

Tumor thickness of SCCs was measured by multiplying the number of axial T2WI slices where the lesion was visible by the slice thickness. Mean tumor thickness of SCCs for high-grade and low-grade group was 22.2 mm and 21.4 mm, respectively. Median tumor volume of SCCs for high-grade and low-grade group was 4.85 cm^3^ and 4.15 cm^3^ (Table 2). No statistical difference has been accessed in either tumor thickness or tumor volume between above two subgroups (*P* = 0.812 and 0.5, respectively).

### Univariate and multi-parametric models in predicting tumor histological grade

**Fig. 3:**
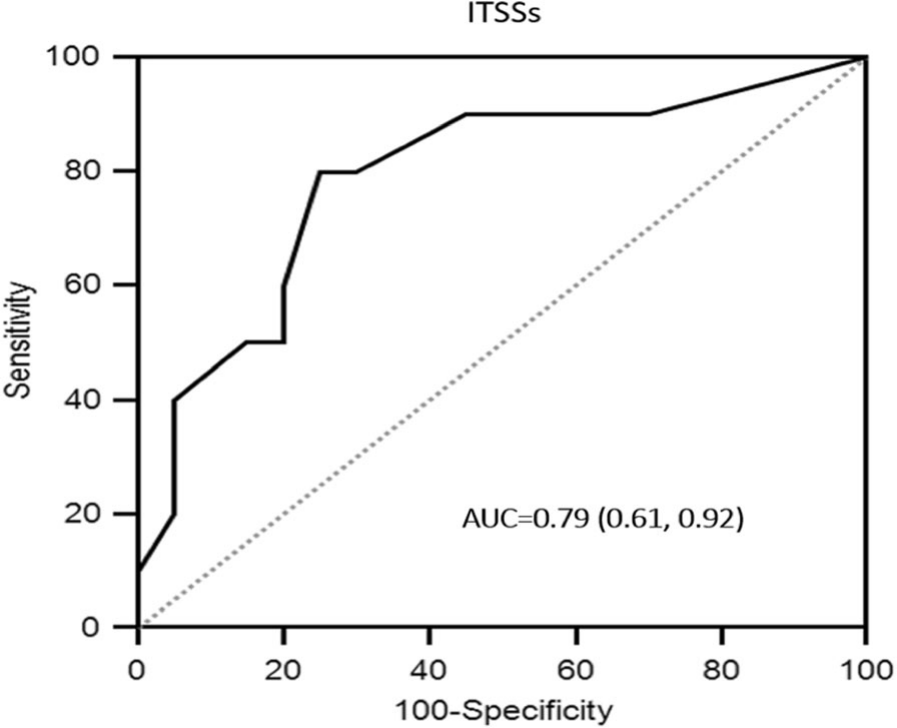
Receiving operating characteristic (ROC) analysis showed the capability of ITSSs in predicting oral tongue cancer histological grade, with an area under the curve of 0.79 (0.61,0.92)

**Fig. 4:**
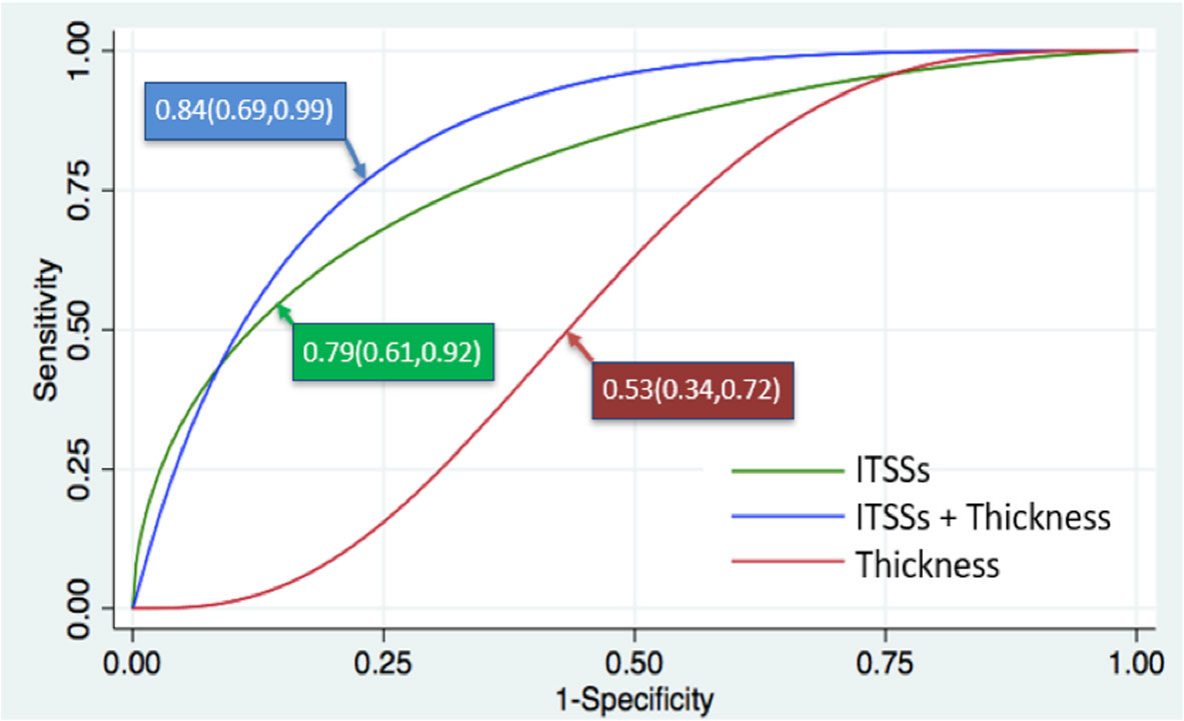
Multi-parametric model using combination of ITSSs and tumor thickness could greatly improve the predictive capability, yielding higher predictive efficiency in comparison with single variable, an area under the curve of 0.84(0.69,0.99)

**Fig. 5:**
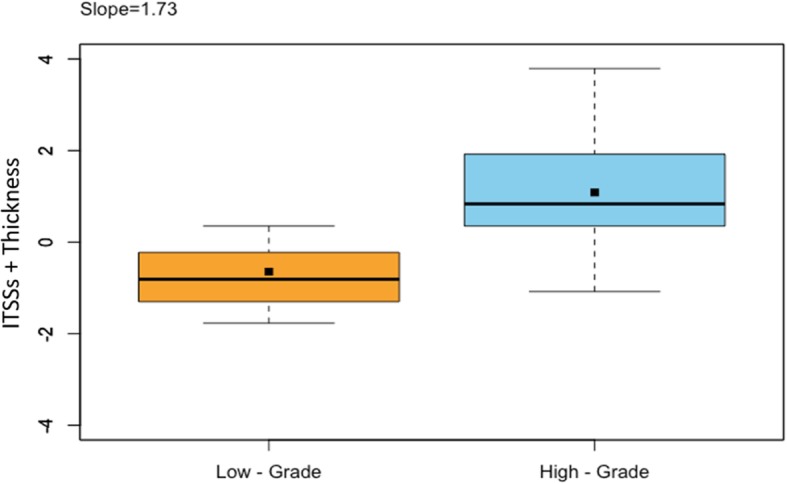
Box plots of multi-parametric model using ITSSs and tumor thickness between low-grade and high-grade groups. The discrimination slope was calculated as the difference of mean predictive scores between low-grade and high-grade groups. Solid dots indicated mean value

To investigate the capability of single variable in predicting tumor histological grade, six parameters including age, tumor thickness, tumor volume, ITSSs, ITSS score and ITSS ratio were selected. ROC analysis showed three ITSS related parameters all performed well in predicting tumor histological grade. The parameter ITSSs was superior to the other two with an area under ROC curve of 0.79, whereas area under ROC curve for ITSS ratio was 0.70 (Fig. 3, Table 3). For access of the combined effect of different parameters on prediction of tumor histological grade, multi-parametric models have been performed. Since ITSSs performed best in single variable ROC analysis, the combination of ITSSs with age, tumor thickness and tumor volume was performed respectively. Our results showed the combination of ITSSs and tumor thickness (2-variable model) greatly improved the predictive capability in comparison with univariate approach, yielding the best performance with the area under ROC curve of 0.84 (0.69–0.99) (Fig. 4, Table 3). Moreover, box plots demonstrated the combination of ITSSs and tumor thickness (2-variable model) allowed low- and high-grade tumors well differentiated (Fig. 5). Inter-rater reliability analysis showed almost perfect reproducibility for above variables. Intra-class correlation coefficient for tumor thickness, tumor volume, ITSSs was 0.99, 0.98, 0.99, respectively. Cohen k for ITSS score was 0.98.

**Table 3:** Predictive efficiency of single or multiparametric models

One variable	c-stastics	Multiple variables	c-stastics
Age	0.42(0.21,0.64)	ITSSs + age	0.79(0.60,0.97)
Volume	0.60(0.41,0.77)	ITSSs + Volume	0.84(0.66,1.00)
Thickness	0.53(0.34,0.72)	ITSSs + thickness	0.84(0.69,0.99)
ITSSs	0.79(0.61,0.92)		
ITSS score	0.73(0.54,0.88)		
ITSS ratio(%)	0.70(0.51,0.85)

### Pre- and post-contrast SWI

**Fig. 6:**
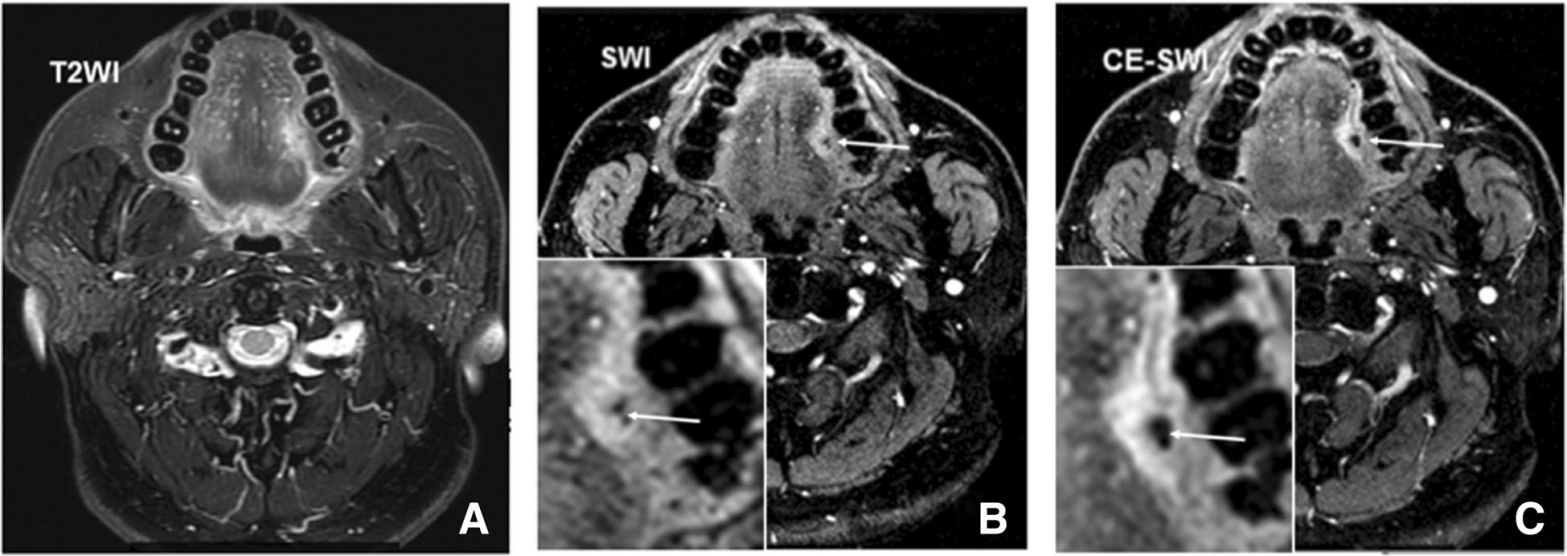
**a**-**c** Representative images showed a low-grade SCC on left oral tongue (**a**) A slightly hyperintense lesion was shown on T2WI. **b** Hyperintense lesion and dot-like ITSSs within the tumor (arrowhead) were seen on SWI. **c** Contrast-enhanced axial SWI showed the internal structure more clearly than non-contrast SWI images (arrowhead)

To further understand the effects of CE-SWI, five of 30 subjects were performed both pre- and post-CE SWI in this study. Representative images showed tumor lesion with obvious enhancement in comparison with non contrast-enhanced SWI images. Moreover, contrast-enhanced axial SWI showed the internal structure more clearly than non-contrast SWI images (Fig. 6). The CNR for pre-contrast SWI and post-contrast SWI was 4.1 ± 1.7 and 7.0 ± 2.5 respectively (values represent the mean ± SD).

## Discussion

Preoperative MR imaging is very important for oral tongue cancer evaluation. MRI could help define tumor extent and detect cervical metastasis due to its excellent soft-tissue contrast. Preoperative tumor TNM or histological grade evaluation is still with great challenge. Non-specific signal characteristics were seen for OTSCC on preoperative MRI (hypointense in T1WI, hyperintense in T2WI, and significantly enhanced in CE-T1WI). Numerous studies working on tumor thickness and volume have found out tumor thickness on MRI had high coincidence with that on histopathology [31, 32]. Meanwhile, tumor thickness on MRI was a great predictive parameter for occult lymph node metastasis [10–12]. Although methods for tumor thickness measurement varied, three-dimensional measurement of tumor thickness was important for oral cancer treatment. In our study, we accessed the tumor thickness by counting slice numbers where the lesion was visible and then multiplying the slice numbers by the slice thickness. We applied this method instead of measurement on sagittal images since marginal tumor with the diameter less than 1 cm might be hard to access in sagittal view. In our study, no statistic difference was shown between low- and high-grade group either in tumor thickness or in tumor volume.

In this study, we applied SWI in OTSCC to facilitate its preoperative prediction of tumor histological grade. SWI was a fairly new technique having advantages in tumor diagnosis and grading. It could maximize the sensitivity to susceptibility effects by combining a high-resolution, fully flow-compensated, 3D gradient-echo sequence with filtered phase information to enhance the contrast in magnitude images [17]. SWI can noninvasively visualize more internal characteristics in tumors and therefore to improve the visibility of tumors and be helpful for depicting hemorrhage, calcification and increased vascularity, which may reflect tumor grade [27, 29]. ITSS score was a semi-quantitative measurement on SWI. Our results showed mean ITSS score was greatly higher in high-grade tumors than that in low-grade OTSCCs. Value of ITSSs and ITSS score in grading gliomas has been described in many studies [19, 22–24], linear or dot-like ITSSs were more frequently observed in high-grade gliomas than in low-grade ones, so that high-grade gliomas got higher ITSS scores. Considering effect of tumor size on ITSS score, we defined ITSS ratio in this study. It was the ratio of ITSSs to lesion involving area, representing for the ITSSs in unit area. Univariate ROC analysis showed among the six selected parameters, three ITSS related parameters (ITSSs, ITSS score, ITSS ratio) performed well in predicting tumor histological grade while tumor thickness and tumor volume did not. ITSSs did the best performance in predicting tumor histological grade, with the area under ROC curve of 0.79. To further access the combined effect of multi-parameters, several multi-parametric models have been tried. Our data demonstrated that although tumor thickness or tumor volume was not strong enough to be a predictive parameter, the combination use with ITSSs exactly improve the predictive capability of tumor histological grade than using ITSSs only. Multi-parametric model using combination of ITSSs and tumor thickness yielded an area under ROC curve of 0.84(0.69–0.99) and allowed great differentiation between different histological groups. Similar results have been reported in recent study working on imaging parameters from SWI, diffusion and perfusion to improve differential diagnosis primary central nervous system lymphomas (PCNSLs) and glioblastomas [25]. ITSSs only yielded an area under ROC curve of 0.753 for differentiation PCNSLS from glioblastomas, while the combination use of ITSSs and ADC mean value or ITSSs and relative regional blood volume (rCBV) mean value would greatly increase the probability for differentiation, and the three-parameter model containing ITSSs, ADC mean value and rCBV performed best. Results form both studies presented superiority of SWI in depiction of intratumoral characteristics. Increased vascularity usually implied a higher tumor grade, as neovascularity was a reflection of fast tumor growth. Malignant tumors usually relied on newly developed vasculature in order to survive or sustain high rates of proliferation, and they are generally associated with increased microvessel density, high vascularity, and cellularity [33]. Semi-quantitative SWI parameters were valuable in tumor differentiation and tumor histological grade prediction, but regarding multiple influence factors such as susceptibility effect and image quality of SWI images, multi-parametric models containing SWI and other parameters would improve accuracy in the area of tumor grade prediction and differentiation.

To further understand the effects of CE-SWI, five of 30 subjects were performed on CE-SWI. Similar to many other studies [34–36], tumors exhibited obvious contrast enhancement on CE-SWI along with ITSSs clearer post contrast agent administration. Admittedly, CE-SWI could help to distinguish hemorrhage from abnormal venous vasculature since blood vessels would change signal intensities whereas hemorrhage would not [37, 38]. However, in our limited subjects with CE-SWI, none of the signal changes were observed in this way and therefore requiring studies with a larger population to be conducted.

Our study had several limitations. One major limitation is that only single axial slice used per patient for SWI analysis may lead to selection bias. In addition, due to small numbers of subjects with high-grade SCCs in our study, for statistic purpose, SCCs were only sub-grouped subjects with SCCs into high- and low-grade groups. Regarding SWI technique, it was noted that paramagnetic object would be enlarged due to “amplified blooming effect” [39], and ITSS might be affected to some degree. Therefore, quantitative method like quantitative susceptibility measurement might be a potential choice for this application. However, our study is the first report of SWI application in OTSCC. Additionally, we did no correlation for scales when using MRI parameters to predict historical grade. Tumor thickness maybe underestimated by measurement of multiplying the number of axial T2WI slices by the slice thickness.

## Conclusions

In conclusion, SWI was superior in depiction of internal characteristics of OTSCCs, which would potentially provide more diagnostic information. Multi-parametric model using combination of ITSSs and tumor thickness would be a promising model for tumor histological grade prediction.

## Abbreviations

CE: Contrast-enhanced
CE-SWI: Contrast-enhanced SWI
CNR: Contrast-to-noise ratio
DOI: Depth of invasion
FSE: Fast-spin-echo
ITSSs: Intratumoral susceptibility signal intensities
MRI: Magnetic resonance imaging
OCC: Oral cavity cancer
OTSCC: Oral tongue squamous cell carcinoma
PCNSL: Primary central nervous system lymphoma
rCBV: Relative regional blood volume
ROC: Receiver operating characteristic
ROI: Region of interest
SCC: Squamous cell carcinoma
SD: Standard deviation
SI: Signal intensity
SWI: Susceptibility weighted imaging
T1WI: T1-weighted imaging
T2WI: T2-weighted imaging
TNM: Tumor Node Metastasis
TT: Tumor thickness
